# Therapeutic Value of Drugs Granted Accelerated Approval or Conditional Marketing Authorization in the US and Europe From 2007 to 2021

**DOI:** 10.1001/jamahealthforum.2022.2685

**Published:** 2022-08-19

**Authors:** Kerstin N. Vokinger, Aaron S. Kesselheim, Camille E. G. Glaus, Thomas J. Hwang

**Affiliations:** 1 Program on Regulation, Therapeutics, and Law, Division of Pharmacoepidemiology and Pharmacoeconomics, Brigham and Women’s Hospital, Harvard Medical School, Boston, Massachusetts; 2 Institute of Law, University of Zurich, Zurich, Switzerland; 3 Cancer Innovation and Regulation Initiative, Lank Center for Genitourinary Oncology, Dana-Farber Cancer Institute, Boston, Massachusetts; 4 Division of Urological Surgery, Brigham and Women's Hospital, Harvard Medical School, Boston, Massachusetts

## Abstract

**Importance:**

The number of drugs approved through the accelerated approval or conditional marketing authorization pathways has increased with unclear evidence of their therapeutic value.

**Objectives:**

To assess the therapeutic value of drug indications granted accelerated approval in the US or conditional marketing authorization in the European Union (EU) overall and for cancer indications.

**Design, Setting, and Participants:**

This cohort study used the public databases of the US Food and Drug Administration and the European Medicines Agency to identify all drugs (initial and supplemental indications) granted accelerated approval in the US or conditional marketing authorization (initial indications only) in the EU between January 1, 2007, and December 31, 2021. Therapeutic value ratings were obtained from national health authorities in Germany, France, and Canada.

**Main Outcomes and Measures:**

Descriptive statistics were used to assess the proportion of accelerated approvals and conditional marketing authorizations overall and for cancer vs noncancer indications rated as having high added therapeutic value.

**Results:**

The cohort included 146 drug indications (94 first indications, 52 supplemental indications) in the US and 58 (all first indications) in the EU. Most drugs were approved for cancer (122 [83.6%] in the US; 40 [69.0%] in the EU). Therapeutic value ratings were available for 90 drug indications (61.6%) in the US and 56 (96.6%) in the EU. Overall, 35 drug indications granted accelerated approval (38.9%) and 21 granted conditional marketing authorization (37.5%) had high added therapeutic value in the US and EU, respectively, at the time of approval. The proportions of indications rated as having high added therapeutic value were 36.0% (27 of 75) for cancer vs 53.3% (8 of 15) for noncancer indications in the US and 30.8% (12 of 39) for cancer vs 52.9% (9 of 17) for noncancer indications in the EU.

**Conclusions and Relevance:**

In this cohort study, among new drug indications approved through the accelerated approval or conditional marketing authorization pathways in the US and Europe from 2007 to 2021, 38.9% and 37.5%, respectively, demonstrated high therapeutic value. A substantially lower proportion of cancer indications than noncancer indications were rated as having high therapeutic value. Policy makers and regulators should increase enforcement of timely postapproval study completion for drugs qualifying for these pathways.

## Key Points

**Question:**

What is the therapeutic value of new drug indications granted accelerated approval or conditional marketing authorization in the US and Europe?

**Findings:**

In this cohort study of 146 drugs, 39% of indications granted accelerated approval and 38% granted conditional marketing authorization were rated as having high added therapeutic value. This proportion was lower for cancer indications than for noncancer indications.

**Meaning:**

The findings suggest that regulators should enforce timely postapproval study completion and review the validity of surrogate measures used to support accelerated approvals.

## Teaser

This cohort study assesses the therapeutic value of drugs granted accelerated approval or conditional marketing authorization in the US and European Union from 2007 to 2021.

## Introduction

The US Food and Drug Administration (FDA) created the accelerated approval pathway in 1992 to speed approval of drugs for serious diseases on the basis of surrogate end points “reasonably likely to predict clinical benefit.”^1^ The use of accelerated approval has increased over the past years, particularly for cancer drugs.^2^ In 2020, 53% of cancer drugs were approved by the FDA through accelerated approval.^3^ The comparable pathway in the European Union (EU) is conditional marketing authorization, which was implemented in 2006.^4^ The European Medicines Agency (EMA) only grants conditional marketing authorization to first (and not supplemental) indications.^5^ Another notable difference is the time limitation of conditional marketing authorization, which must be renewed annually with an application and interim report on outstanding study obligations.^5^

In recent years, there has been considerable controversy about accelerated approval. One key example was the FDA’s accelerated approval of aducanumab, an amyloid beta-targeted monoclonal antibody approved after clinical trials showed unclear therapeutic value for patients.^6^ In contrast to the FDA, the EMA concluded that the benefits of aducanumab did not outweigh its risks and recommended refusing marketing authorization. The manufacturer eventually withdrew its application in April 2022.^7^

Prior studies^8,9^ have found that fewer than one-third of new drugs approved by the FDA and EMA were rated by independent health technology assessment organizations as having a high added therapeutic value compared with existing treatments. The results also indicated that drugs approved through expedited pathways were more likely than nonexpedited drugs to be rated as having a high therapeutic value.^8,9^ However, the added therapeutic value of drugs approved through accelerated approval and conditional marketing authorization pathways and differences between the FDA and EMA programs are unclear. In this cohort study, we assessed the therapeutic value of drug indications granted accelerated approval or conditional marketing authorization, focusing on differences between those indicated for cancer vs noncancer conditions.

## Methods

We used the FDA’s and EMA’s public databases to identify all drugs (for initial and supplemental indications) granted accelerated approval in the US or conditional marketing authorization (for initial indications only) in the EU between January 1, 2007, and December 31, 2021.^10,11^ We also identified all drugs that were granted temporary authorization in Switzerland, an approval pathway equivalent to the conditional marketing authorization that was introduced in 2019.^12^ This study followed the Strengthening the Reporting of Observational Studies in Epidemiology (STROBE) reporting guideline for cohort studies. The study was deemed exempt from institutional review board approval according to 45 CFR §46.102 because it used public, nonidentifiable data and did not constitute human participants research.

We obtained therapeutic value ratings from German (Federal Joint Committee), French (National Authority for Health), and Canadian (Human Drug Advisory Panel) health technology assessment agencies, which assess added therapeutic value of new medicines compared with existing therapies.^13,14,15^ As in prior studies,^8,9^ ratings of moderate or greater added therapeutic value by at least 1 agency were defined as high. We also reviewed any reevaluations of ratings conducted and published as of May 2022.

### Statistical Analysis

Using descriptive analysis, we assessed the proportion of accelerated approvals and conditional marketing authorizations overall and for cancer vs noncancer indications that were rated as having high therapeutic added value. Analyses were conducted using R, version 4.1.0 (R Project for Statistical Computing).

## Results

From 2007 to 2021, 146 drug indications (94 first indications, 52 supplemental indications) were granted accelerated approval by the FDA and 58 (all first indications) received conditional marketing authorization by the EMA, with use of both pathways increasing over time (Figure). Most of these approved indications targeted cancer (122 [83.6%] in the US; 40 [69.0%] in the EU). In Switzerland, 13 drug indications (all first indications only) were granted temporary authorization. As of December 2021, 48 accelerated approvals (32.9%) and 21 conditional marketing authorizations (35.0%) were converted to regular approval.

**Figure.**
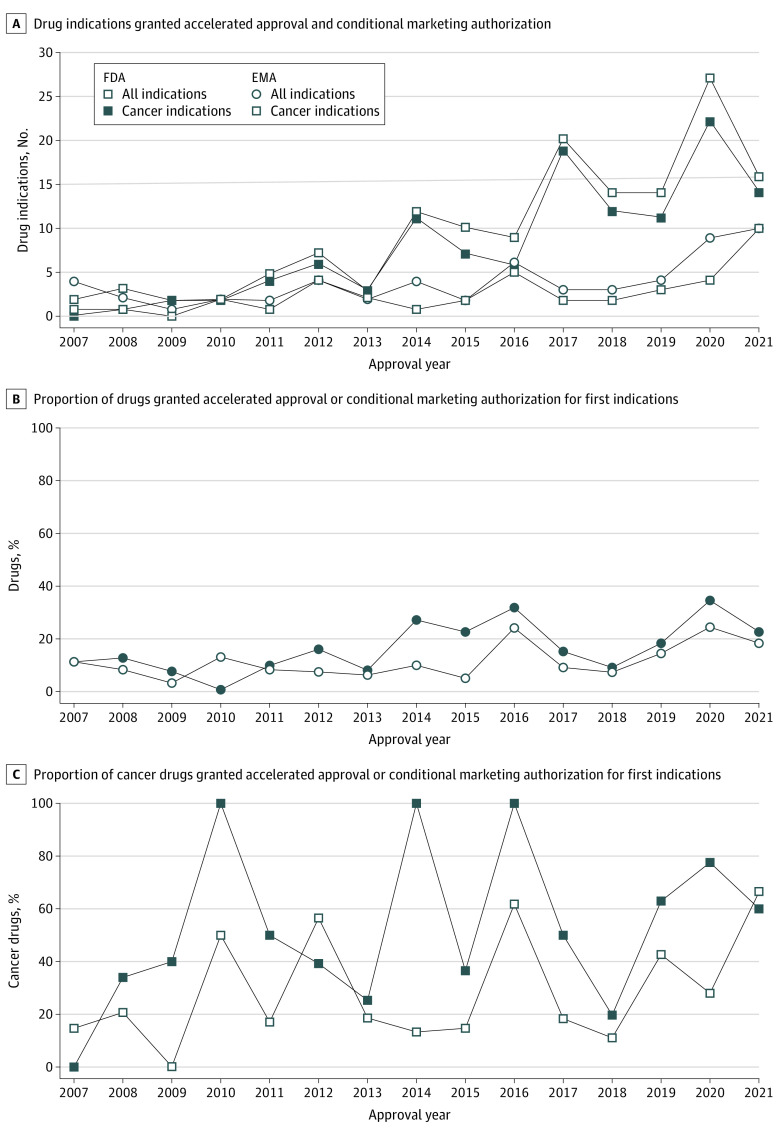
Trends in Accelerated Approval and Conditional Marketing Approvals From 2007 to 2021 A, Total drug indications granted accelerated approval and conditional marketing authorization in the US and Europe. B, Proportion of drugs (first indications) granted accelerated approval or conditional marketing authorization among total approved drugs. C, Proportion of cancer drugs (first indications) granted accelerated approval or conditional marketing authorization among total approved cancer drugs. EMA indicates European Medicines Agency; FDA, US Food and Drug Administration.

Of the 146 drug indications granted accelerated approval, 33 (22.6%) were also conditionally approved in the EU. Fifty-four (37%) were granted regular approval, 2 (1.4%) were approved through an expedited pathway, 5 (3.4%) were refused, 3 (2%) had applications withdrawn, and 49 (33.6%) were still under review or had not been submitted to the EMA. Of the 58 drug indications conditionally approved in the EU, most (33 [56.9%]) were also granted accelerated approval; 14 (24.1%) were granted at least 1 expedited review program (but not accelerated approval), 4 (6.9%) were approved through the regular pathway, 2 (3.4%) were refused, and 5 (8.7%) were still under review or had not been submitted to the FDA by the end of our data collection process.

In the US, the number of indications granted accelerated approval increased after 2013 (Figure). Almost all supplemental indications (50 of 52 [96.2%]) were approved for treatment of cancer, with 2 drugs (nivolumab and pembrolizumab) accounting for more than half of these supplemental approvals (26 of 52 [50.0%]). In the EU, a slower annual increase in drugs granted conditional marketing authorization was observed (Figure).

Therapeutic value ratings were available for 90 drug indications (61.6%) in the US and 56 in the EU (96.6%). Thirty-five accelerated approvals (38.9%) and 21 conditional marketing authorizations (37.5%) were rated as having high added therapeutic value in the US and EU, respectively, at the time of approval (Table). The proportions of drug indications rated as having high added therapeutic value were 36.0% (27 of 75) for cancer vs 53.3% (8 of 15) for noncancer indications in the US and 30.8% (12 of 39) for cancer vs 52.9% (9 of 17) for noncancer indications in the EU. Therapeutic value ratings were available for 11 drug indications (84.6%) in Switzerland, with 1 drug indication (9.0%) rated highly.

**Table.** Characteristics of Drug Indications Granted Accelerated Approval and Conditional Marketing Authorization by the FDA and EMA

Therapeutic area	Drug indications, No. (%)			
	Accelerated approval	Conditional marketing authorization	Available therapeutic value rating	
			FDA	EMA
All	146 (100)	58 (100)	90 (100)	56 (100)
Antineoplastic	122 (83.6)	40 (69.0)	75 (83.3)	39 (69.6)
Anti-infective	9 (6.2)	8 (13.8)	8 (8.9)	7 (12.5)
Blood and cardiovascular	8 (5.5)	2 (3.4)	5 (5.6)	2 (3.6)
Musculoskeletal	5 (3.4)	6 (10.4)	0	6 (10.7)
Alimentary and metabolism	2 (1.4)	1 (1.7)	2 (2.2)	1 (1.8)
Systemic hormonal preparations	0	1 (1.7)	0	1 (1.8)

Abbreviations: EMA, European Medicines Agency; FDA, US Food and Drug Administration.

When including reevaluations of therapeutic value ratings based on postapproval evidence until December 2017, 36 drugs in the US (40.0%) and 22 in the EU (39.2%) had high added therapeutic value. When focusing only on first indications granted accelerated approval in the US or conditional marketing authorization in the EU, 37.7% (23 of 61) in the US and 37.5% (21 of 56) in the EU were rated as having high therapeutic value. Of drugs with initial approvals for cancer indications, 33.3% (16 of 48) in the US had high added therapeutic value compared with 30.8% (12 of 39) in the EU.

## Discussion

From 2007 to 2021, the number of new drug indications granted accelerated approval in the US or conditional marketing authorization in the EU increased, with many of these indications approved for the treatment of cancer. However, only 38.9% of drugs granted accelerated approval in the US and 37.5% granted conditional marketing authorization in the EU were rated as providing moderate or greater therapeutic value compared with existing therapies. This proportion was substantially lower for cancer than for other indications.

The value assessments by the German, French, and Canadian health technology assessment agencies serve as a basis for price negotiations and reimbursement in those countries.^16,17,18^ In addition, the value scores that the agencies assign may help guide physicians and patients in treatment selection.^19^ In December 2021, the EU adopted a new regulation to harmonize value assessment of new medicines across the EU starting in 2025, with cancer drugs to be among the first therapies subjected to this centralized assessment.^20^ However, the increasing use of accelerated and conditional approval pathways may complicate the work of these health agencies charged with assessing the value of new therapies. Prior studies have highlighted the inherently limited and uncertain evidentiary basis on which many drugs are granted accelerated approval and conditional marketing authorization, with questions about the validity of the surrogate measures and biomarkers used to support such approvals.^16,21^

Reevaluations of therapeutic value ratings resulted in only 1 indication for the US and EU that was initially considered to have low therapeutic value to be subsequently rated as providing high therapeutic value (ceritinib for treatment of advanced non–small cell lung cancer). This finding is consistent with prior literature indicating that only a fraction of postapproval trials demonstrates superior efficacy compared with outcomes of pivotal trials.^22,23,24,25^ Our study findings that many drugs granted accelerated approval or conditional marketing authorization did not appear to offer high added therapeutic value at the time of approval underscore the importance of timely completion of required postapproval studies for drugs and of ensuring that these confirmatory studies test clinically meaningful end points. Our results also support ongoing legislative efforts to reform the accelerated approval pathway.^26^ The Food and Drug Amendments of 2022, passed by the US House of Representatives in June 2022, would allow the FDA to require initiation of confirmatory studies before accelerated approval, setting of enrollment targets, and biannual reporting of study progress. The bill would also codify an expedited withdrawal procedure that can be triggered if the sponsor fails to conduct the required postapproval study, the confirmatory study fails to confirm benefit, other evidence demonstrates the product is not safe and effective, or if the sponsor disseminates false or misleading promotional materials.

### Limitations

This study has limitations. Therapeutic value ratings were not available for drugs receiving accelerated approval in the US that were not approved in Europe or Canada. A prior study^27^ found that drugs approved only in the US and not also in Europe were more likely than drugs approved in both jurisdictions to offer low therapeutic value.

## Conclusions

In this cohort study, although an increasing number of new drug indications were approved through the accelerated approval and conditional marketing authorization pathways from 2007 to 2021, most were rated as not providing high added therapeutic value at the time of approval. In some cases, these low ratings might be due to the uncertain nature of the data supporting the drugs approved through these pathways. Policy makers could consider increasing enforcement of timely postapproval study completion and reviewing the validity of surrogate measures used in these approvals.

## References

[1] Food and Drug Administration. Accelerated approval. Accessed June 21, 2022. https://www.fda.gov/patients/fast-track-breakthrough-therapy-accelerated-approval-priority-review/accelerated-approval

[2] Darrow JJ, Avorn J, Kesselheim AS. FDA approval and regulation of pharmaceuticals, 1983-2018. JAMA. 2020;323(2):164-176. doi:10.1001/jama.2019.20288 31935033

[3] Gyawali B, Ross JS, Kesselheim AS. Fulfilling the mandate of the US Food and Drug Administration’s accelerated approval pathway: the need for reforms. JAMA Intern Med. 2021;181(10):1275-1276. doi:10.1001/jamainternmed.2021.4604 34254981

[4] European Medicines Agency. Conditional marketing authorisation. Accessed June 21, 2022. https://www.ema.europa.eu/en/human-regulatory/marketing-authorisation/conditional-marketing-authorisation

[5] Mehta GU, de Claro RA, Pazdur R. Accelerated approval is not conditional approval: insights from international expedited approval programs. JAMA Oncol. 2022;8(3):335-336. doi:10.1001/jamaoncol.2021.6854 35050302

[6] Alexander GC, Knopman DS, Emerson SS, . Revisiting FDA approval of aducanumab. N Engl J Med. 2021;385(9):769-771. doi:10.1056/NEJMp2110468 34320282 PMC11694499

[7] European Medicines Agency (EMA). Aduhelm: withdrawal of the marketing authorisation application. Accessed June 21, 2022. https://www.ema.europa.eu/en/medicines/human/withdrawn-applications/aduhelm

[8] Hwang TJ, Ross JS, Vokinger KN, Kesselheim AS. Association between FDA and EMA expedited approval programs and therapeutic value of new medicines: retrospective cohort study. BMJ. 2020;371:m3434. doi:10.1136/bmj.m3434 33028575 PMC7537471

[9] Vokinger KN, Hwang TJ, Glaus CEG, Kesselheim AS. Therapeutic value assessments of novel medicines in the US and Europe, 2018-2019. JAMA Netw Open. 2022;5(4):e226479. doi:10.1001/jamanetworkopen.2022.6479 35385091 PMC8987900

[10] Food and Drug Administration. Drugs@FDA. Accessed June 21, 2022. https://www.accessdata.fda.gov/scripts/cder/daf/

[11] European Medicines Agency. Medicines. Accessed June 21, 2022. https://www.ema.europa.eu/en/medicines

[12] Swiss Confederation. Federal Act on Medicinal Products and Medical Devices. Accessed July 18, 2022. https://www.fedlex.admin.ch/eli/cc/2001/422/en

[13] Federal Joint Committee. The benefit assessment of medicinal products in accordance with the German Social Code, book 5 (SGB V), section 35a. Accessed June 21, 2022. https://www.g-ba.de/english/benefitassessment/

[14] Haute Autorité de Santé. News. Accessed June 21, 2022. https://www.has-sante.fr/jcms/fc_2876008/fr/medicament

[15] Government of Canada. Human Drug Advisory Panel. Accessed June 21, 2022. https://www.pmprb-cepmb.gc.ca/view.asp?ccid=478

[16] Ludwig WD. Zehn Jahre AMNOG—Rückblick und Ausblick aus Sicht der Arzneimittelkommission der deutschen Ärzteschaft. In: Schwabe U, Ludwig WD, eds. Arzneiverordnungs-Report 2020. Springer; 2020

[17] Haute Autorité de Sante. Transparency Committee. Accessed June 21, 2022. https://www.has-sante.fr/jcms/c_1729421/en/transparency-committee

[18] Government of Canada. Patented Medicine Prices Review Board: frequently asked questions. Accessed June 21, 2022. http://www.pmprb-cepmb.gc.ca/about-us/frequently-asked-questions

[19] Institute for Quality and Efficiency in Health Care. Early benefit assessment of drugs: effects on healthcare in Germany. Accessed June 21, 2022. https://www.iqwig.de/presse/pressemitteilungen/pressemitteilungen-detailseite_51008.html

[20] Hwang TJ, Vokinger KN. New EU regulation on health technology assessment of cancer medicines. Lancet Oncol. 2022;23(2):e58. doi:10.1016/S1470-2045(22)00008-0 35114127

[21] Haas A, Mayer T, Tebinka-Olbrich A, Blindzellner M, Beggerow E, Nickel A. Beschleunigte Zulassung von Arzneimitteln: Herausforderungen für Patient:innen, Datenqualität und faire Preise. In: Schröder H, Thürmann P, Telschow C, Schröder M, Busse R, eds. Arzneimittel-Kompass 2021. Springer; 2021. doi:10.1007/978-3-662-63929-0_8

[22] Beaver JA, Howie LJ, Pelosof L, . A 25-year experience of US Food and Drug Administration accelerated approval of malignant hematology and oncology drugs and biologics: a review. JAMA Oncol. 2018;4(6):849-856. doi:10.1001/jamaoncol.2017.5618 29494733

[23] Gyawali B, Hey SP, Kesselheim AS. Assessment of the clinical benefit of cancer drugs receiving accelerated approval. JAMA Intern Med. 2019;179(7):906-913. doi:10.1001/jamainternmed.2019.0462 31135808 PMC6547118

[24] Pease AM, Krumholz HM, Downing NS, Aminawung JA, Shah ND, Ross JS. Postapproval studies of drugs initially approved by the FDA on the basis of limited evidence: systematic review. BMJ. 2017;357:j1680. doi:10.1136/bmj.j1680 28468750 PMC5421452

[25] Gyawali B, Rome BN, Kesselheim AS. Regulatory and clinical consequences of negative confirmatory trials of accelerated approval cancer drugs: retrospective observational study. BMJ. 2021;374:n1959. doi:10.1136/bmj.n1959 34497044 PMC8424519

[26] Hwang TJ, Trinh QD, Tibau A, Vokinger KN. Reforms to accelerated approval of new medicines: long overdue. Lancet. Published online July 11, 2022 doi:10.1016/S0140-6736(22)01276-4 35835129

[27] Larochelle M, Downing NS, Ross JS, David FS. Assessing the potential clinical impact of reciprocal drug approval legislation on access to novel therapeutics in the USA: a cohort study. BMJ Open. 2017;7(2):e014582. doi:10.1136/bmjopen-2016-014582 28179418 PMC5306516

